# Exploring the growth and impact of artificial intelligence in anesthesiology: a bibliometric study from 2004 to 2024

**DOI:** 10.3389/fmed.2025.1595060

**Published:** 2025-06-02

**Authors:** Keke Liu, Weicheng Qiu, Xinping Yang

**Affiliations:** Department of Anesthesiology, Shenzhen Second People’s Hospital, the First Affiliated Hospital of Shenzhen University, Shenzhen, China

**Keywords:** intraoperative monitoring, predictive modeling, VOSviewer, CiteSpace, bibliometric analysis

## Abstract

**Background:**

The integration of artificial intelligence (AI) in anesthesiology is revolutionizing clinical practice by enhancing patient monitoring, improving risk assessment, and enabling personalized anesthetic care. This bibliometric analysis aims to evaluate publication trends, key contributors, and emerging translational pathways in AI research in anesthesiology, with special emphasis on clinical relevance, thematic clustering, and future application prospects.

**Materials and methods:**

Publications related to AI in anesthesiology from 2004 to 2024 were retrieved from the Web of Science Core Collection database, resulting in 658 articles. VOSviewer and CiteSpace were employed for the bibliometric analysis.

**Results:**

AI research in anesthesiology has experienced substantial growth, with a notable surge between 2019 and 2020. The United States leads in both publication volume and citation impact, reflecting its central role in advancing AI-driven innovations. Major journals such as *Anesthesia and Analgesia* and *Anesthesiology* play central roles in disseminating key findings. Keyword and journal cluster analyses revealed three major translational domains: real-time perioperative risk prediction (e.g., hypotension, mortality), AI-assisted ultrasound for regional anesthesia, and intelligent anesthesia monitoring systems. Despite progress, emerging concerns such as model interpretability, patient-centered outcomes, and multimodal data integration remain underexplored.

**Conclusion:**

AI in anesthesiology is entering a phase of rapid interdisciplinary expansion, integrating clinical needs with computational innovation. Future research should prioritize the clinical validation of AI tools, foster stronger collaboration between computer scientists and anesthesiologists, and address unresolved translational gaps such as model interpretability and cross-modal data fusion.

## 1 Introduction

Artificial intelligence (AI) is a branch of computer science focused on simulating and extending human-like intelligent behavior, particularly in areas such as image recognition, natural language processing, language translation, text analysis, and self-learning (1). By leveraging technologies like machine learning and deep learning, AI can process vast amounts of data, identify patterns, make data-driven decisions, and solve complex problems by developing algorithms or models capable of performing predictive tasks without explicit programming instructions (2). Computer vision, a subset of AI, enables machines to interpret and analyze visual data, such as computed tomography images, through the automatic acquisition, processing, and understanding of medical imagery.

AI techniques have shown significant promise in various medical domains, including screening, diagnosis, treatment planning, and patient management across fields such as cardiology, oncology, and vascular surgery (3–7). Anesthesiology, which requires clinical decisions based on multiple continuous real-time variables, stands to benefit particularly from these advancements. With the rise in computing power and the accumulation of clinical databases, AI has demonstrated substantial potential in assessing risks for complications such as intraoperative hypotension, acute kidney injury, and postoperative delirium (8, 9). Additionally, AI-assisted ultrasound technology, powered by computer vision, has made significant strides in anesthesiology. Existing literature in this field can be categorized into several subdomains based on their clinical applications, including depth of anesthesia monitoring, computer vision-guided techniques, prediction of perioperative and postoperative events, anesthesia control, pain management, and operating room logistics (9).

Bibliometrics is both a qualitative and quantitative technique used to analyze academic literature (10). Despite the rapid expansion of AI-related research in anesthesiology, a comprehensive bibliometric analysis of this field remains lacking. With the increasing maturity and deployment of AI technologies in healthcare, there is an urgent need to understand not only their algorithmic development but also the pathways that lead from technical innovation to patient-centered clinical applications. By examining keyword co-occurrence patterns, journal distributions, and research clusters, this study sheds light on how interdisciplinary integration and evolving perioperative demands are shaping the trajectory of AI in anesthesiology.

Moreover, based on the observed gap between current keyword trends and clinical implementation needs, we suggest that domains such as model interpretability, outcome-based validation, and multimodal data integration warrant greater attention in future research.

## 2 Materials and methods

### 2.1 Data source and search strategy

**Figure 1:**
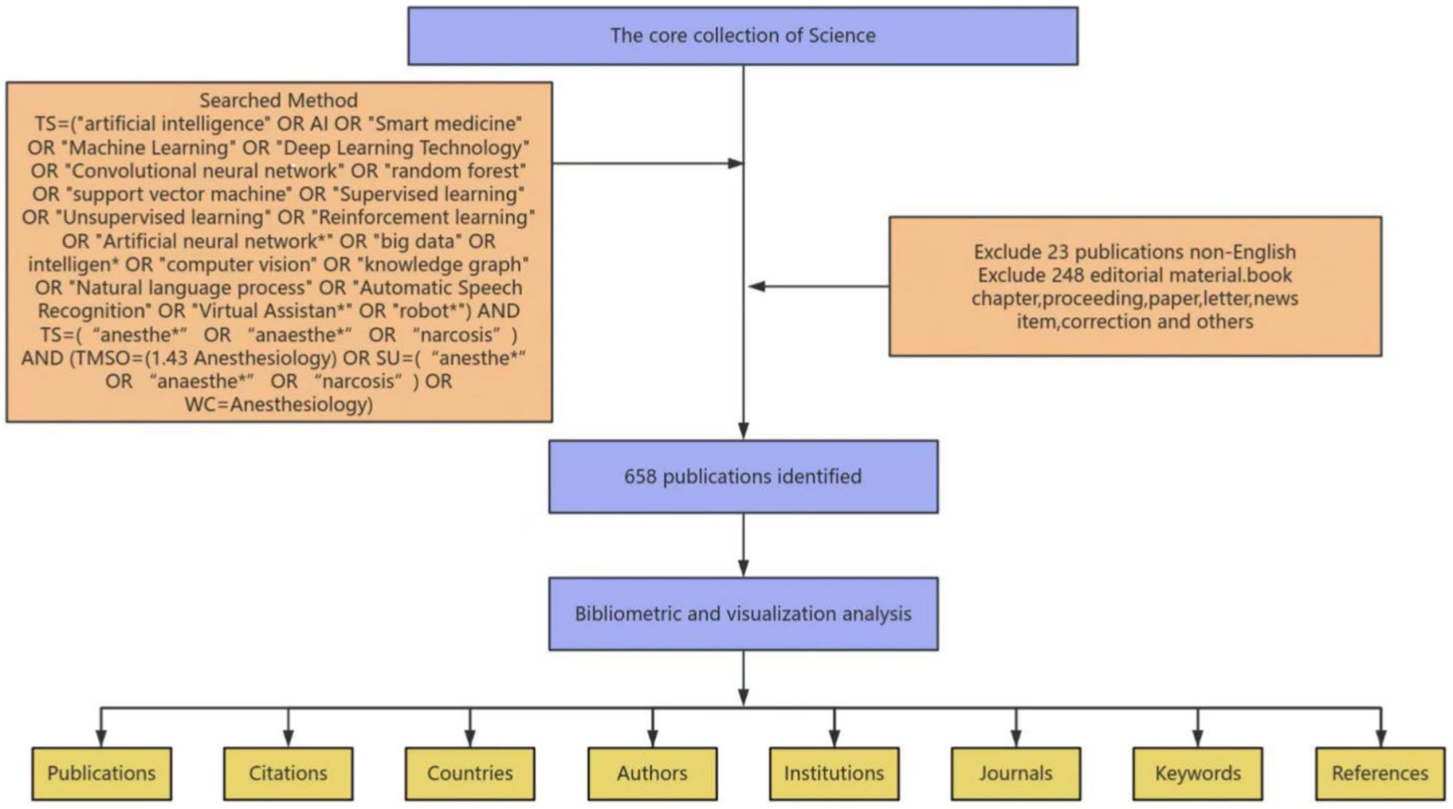
Detailed flowchart steps of the search strategy for publications screening.

All bibliographic data in this article are derived from the Web of Science Core Collection database (11, 12), which covers academic publications in nearly 300 disciplines worldwide. The time span of the bibliometric analysis conducted in this article ranges from January 1, 2004, to September 16, 2024. Figure 1 illustrates the specific steps used in the data retrieval and inclusion processes, along with the search strategy. The retrieval process was performed independently by two researchers, and any disagreements were resolved through discussions with a senior anesthesiologist until a consensus was reached.

### 2.2 Data analysis

After confirming the accuracy of the data, we exported the filtered and optimized raw dataset in plain text file format, including key information such as title, author, keywords, institution, country/region, citations, journal, and publication date. Subsequently, we used Microsoft Office Excel 2021, VOSviewer (version 1.6.18), and CiteSpace (version 6.1.R6) as the main tools for data analysis and visualization.

CiteSpace (13, 14), developed by Chaomei Chen and colleagues, is used for creating network maps of specific domains and extracting key information from research, such as emerging trends, hotspots, and directions. In this study, we used this software to analyze the co-occurrence and clustering of authors, research institutions, and countries in the literature, and simultaneously used VOSviewer (15, 16)—a Java-based literature tracking software developed by Nees Jan van Eck and colleagues in 2010, suitable for visual analysis of various types of data—to analyze the distribution of countries/regions involved, institutional distribution, author collaboration patterns, and the distribution and relationships of keywords.

## 3 Results

### 3.1 Publication and citation analysis

**Figure 2:**
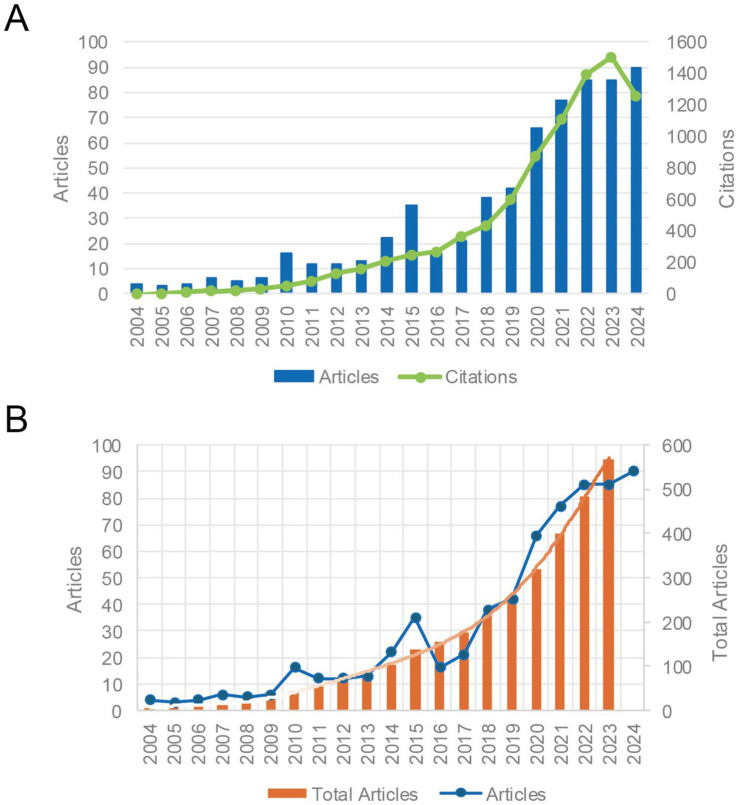
Trends in publications and citations of AI research in anesthesiology (2004–2024). **(A)** Annual publications and citations of AI research in anesthesiology. **(B)** Annual and cumulative publications with polynomial fitting.

Figure 2A shows the trend of publications and citations in the field of AI in anesthesiology from 2004 to 2024. The number of publications has been generally increasing with fluctuations. Meanwhile, citations have steadily risen each year, reaching 1,508 in 2023. Notably, there was a significant surge in the number of publications between 2019 and 2020. By September 2024, 90 articles have already been published in this field, surpassing the annual publication volume of previous years.

As depicted in Figure 2B, Polynomial regression analysis (equation: y = –0.0002x^6^ + 0.0124x^5^ – 0.2872x^4^ + 3.1536x^3^ – 15.872x^2^ + 37.82x – 22.775) predicts sustained growth. An exponential model fitted to the complete 2024 dataset (see Supplementary Figure 1) further corroborates the field’s accelerating trajectory.

### 3.2 Countries/regions analysis

**Figure 3:**
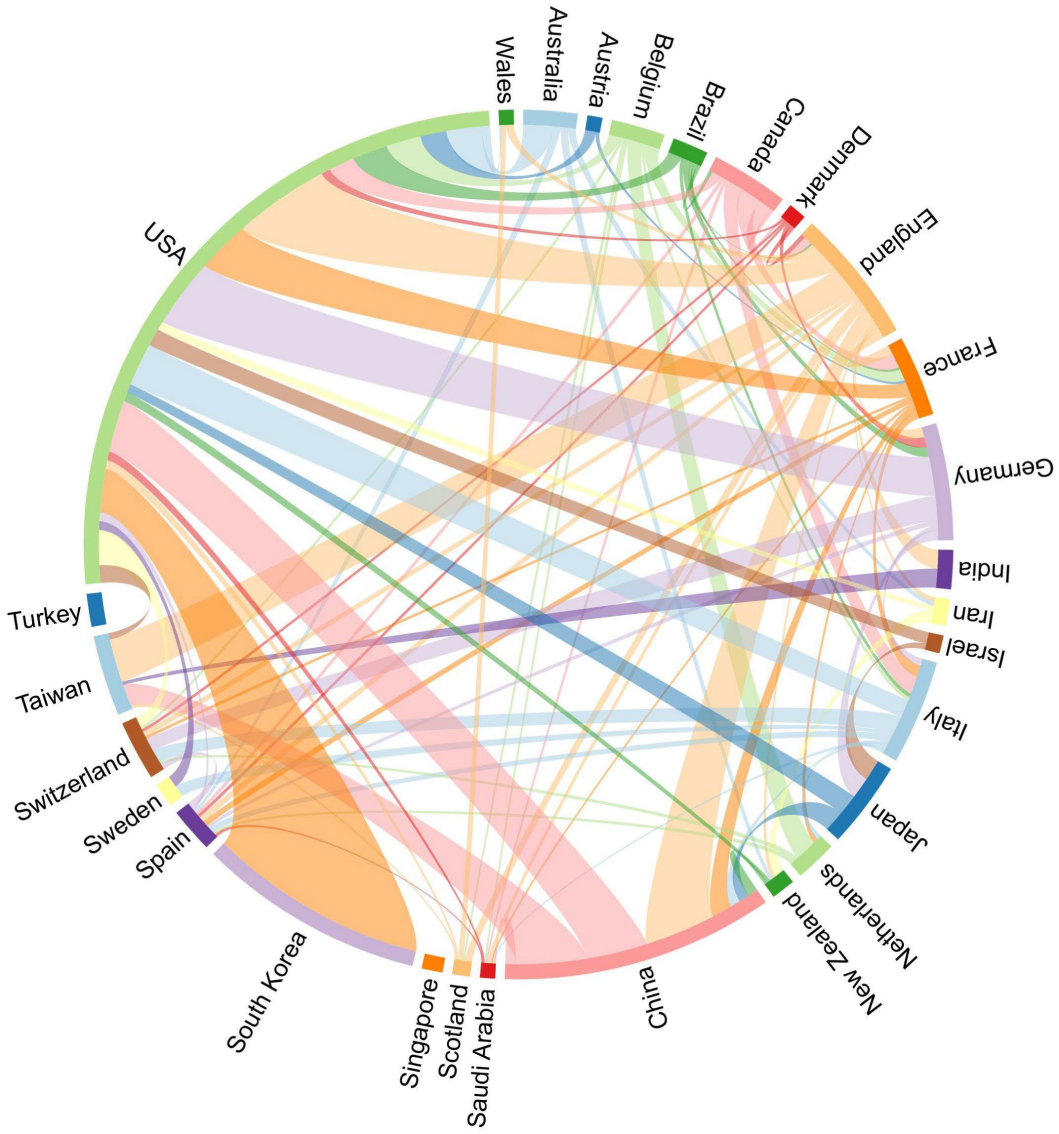
National collaboration network in AI research in anesthesiology.

Table 1 and Figure 3 illustrate the global distribution of publications and citations in AI in anesthesiology. The United States leads in both the number of publications (228) and citations (3,713), indicating its dominant role in this research area. Other significant contributors include South Korea (76 publications, 1,026 citations), China (91 publications, 611 citations), and England (45 publications, 809 citations). Notably, Belgium ranks tenth in citations (342), despite not being among the top 10 for publications, highlighting its high academic influence in the field. VOSviewer analysis reveals that the United States has extensive academic connections with countries like South Korea, China, Germany, and England, reflecting its central position in the field. South Korea, which is not short of publication numbers, has only developed a more obvious cooperative relationship with the United States.

**Table 1:** Top 10 countries/regions in AI research in anesthesiology.

Rank	Countries	Documents	Countries	Total link strength	Countries	Citations
1	United States	228	United States	76	United States	3,713
2	China	91	England	63	South Korea	1,026
3	South Korea	76	France	33	England	809
4	England	45	Italy	28	Germany	692
5	Germany	39	China	26	China	611
6	Italy	35	Taiwan	25	Taiwan	547
7	Japan	29	Germany	23	Canada	496
8	France	27	Canada	22	Netherlands	391
9	Taiwan	27	Switzerland	22	Italy	343
10	Canada	26	Belgium	18	Belgium	342

To better understand the development trajectory of global research efforts in this field, we conducted a time-series analysis of annual publication trends across the top 10 most productive countries (Supplementary Figure 2). The United States has consistently maintained its leadership in publication output throughout the past two decades. Notably, China exhibited a significant surge in publications beginning in 2022, firmly establishing itself as the second most prolific contributor. South Korea maintained a stable and upward trajectory, while England demonstrated a sharp increase in 2024, potentially indicating a strategic pivot toward AI-driven anesthesiology research.

### 3.3 Author analysis

Table 2 records the countries/regions, institutions, and total link strengths of the top 10 authors in terms of publication volume and co-citation frequency in the field of AI in anesthesiology. It can be seen that there are three authors who have published more than 10 papers, namely Shieh, Jiann-Shing from Taiwan (14 papers), Fan, Shou-Zen from Taiwan (13 papers), and Abbod, Maysam F. from England (11 papers). The co-citation relationships of authors show that Myles, Ps from Australia leads with 51 co-citations, followed by Hemmerling, Tm from Canada (43 co-citations) and Liu, Q from China (42 co-citations), This indicates that these authors have a significant academic influence in this field.

**Table 2:** Top 10 authors in AI research in anesthesiology.

Rank	Author	Documents	Total link strength	Countries/regions	Institution	Author	Co-citations	Total link strength	Countries/regions	Institution
1	Shieh, Jiann-Shing	14	41	Taiwan	Yuan Ze University	Myles, Ps	51	172	Australia	Florey Institute of Neuroscience and Mental Health
2	Fan, Shou-Zen	13	41	Taiwan	National Taiwan University	Hemmerling, Tm	43	227	Canada	McGill University
3	Abbod, Maysam F.	11	39	England	Brunel University	Liu, Q	42	219	China	Sun Yat Sen University
4	Hemmerling, Thomas M.	8	20	Canada	McGill University	Purdon, Pl	42	208	United States	Stanford University
5	Lee, Hyung-Chul	7	10	South Korea	Seoul National University Hospital	Schnider, Tw	36	194	Switzerland	Kantonsspital St. Gallen
6	Liu, Quan	7	28	China	Sun Yat Sen University	Bruhn, J	35	157	Netherlands	Radboud University Nijmegen
7	Bai, Sun-Joon	6	23	South Korea	Yonsei University	Shalbaf, R	35	202	Iran	Institute for Cognitive Sciences Studies
8	Choi, Young Deuk	6	17	South Korea	Yonsei University	Bowness, J	33	104	England	University of Oxford
9	Kim, Na Young	6	16	South Korea	Ajou University	Lundberg, Sm	29	128	United States	Microsoft
10	Sessler, Daniel I.	6	16	United States	Cleveland Clinic	Apfel, Cc	28	74	United States	University of California San Francisco

**Figure 4:**
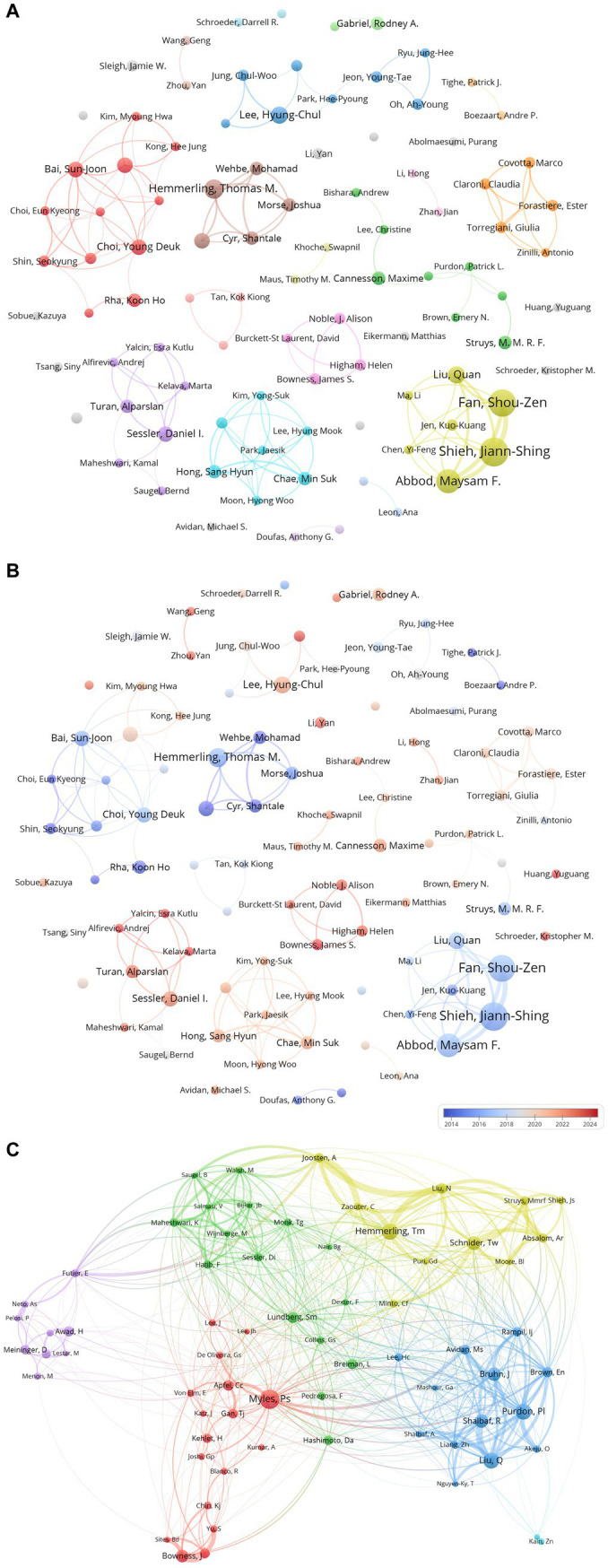
Author collaboration and citation networks in AI research in anesthesiology. **(A)** Co-occurrence network of authors in AI research in anesthesiology (node color represents author clusters; node size indicates co-occurrence frequency; links depict collaborations). **(B)** Author influence and activity trends in AI research in anesthesiology (based on **A**, red indicates increasing influence, blue indicates declining activity). **(C)** Co-citation network of authors in AI research in anesthesiology (node size represents citation frequency).

Figure 4 visualizes author cooperation, showing regional clusters of authors with strong academic connections. The brown and yellow clusters stand out for their high publication volumes, with key authors such as Hemmerling and Fan leading these groups in Figure 4A. It can be seen that the author clusters with high publication volumes in Figure 4A had already started researching in the field of AI in anesthesiology before 2020 and developed obvious cooperative relationships combining the analysis of author cooperation in Figure 4B.

Co-citation analysis in Figure 4C reveals that the yellow cluster represents strong co-citation relationships in anesthesiology, particularly its technological applications in engineering and computer science. The blue cluster focuses on anesthesiology and neurology, while the green cluster delves into healthcare services. The red cluster is centered on anesthesiology and general medicine, and the purple cluster covers obstetrics, surgery, and oncology. Additionally, there is significant overlap in authors’ research areas, especially in anesthesiology, engineering, and computer science.

### 3.4 Institution analysis

Table 3 ranks the top 10 institutions by publication volume and citation frequency. Yonsei University (South Korea) leads with 25 publications, followed by Seoul National University (20 publications). U.S. institutions, including Stanford University (14 publications) and Harvard Medical School (13 publications), also contribute significantly. The top three in citation frequency are Mayo Clinic (456 citations), Yonsei University (409 citations), and Yuan Ze University (367 citations), reflecting strong academic influence from the United States, South Korea, and Taiwan.

**Table 3:** Top 10 institutions in AI research in anesthesiology.

Rank	Institution	Publications	Original country	Institution	Citations	Original country
1	Yonsei University	25	South Korea	Mayo Clinic	456	United States
2	Seoul National University	20	South Korea	Yonsei University	409	South Korea
3	Stanford University	14	United States	Yuan Ze University	367	Taiwan
4	Yuan Ze University	14	Taiwan	Harvard University	354	United States
5	Harvard Medical School	13	United States	National Taiwan University	351	Taiwan
6	National Taiwan University	13	Taiwan	Seoul National University	297	South Korea
7	Cleveland Clinic	11	United States	Brunel University London	240	England
8	Icahn School of Medicine at Mount Sinai	11	United States	University of Ghent	238	Belgium
9	Mayo Clinic	11	United States	University of Groningen	227	Netherlands
10	McGill University	11	Canada	Stanford University	187	United States

**Figure 5:**
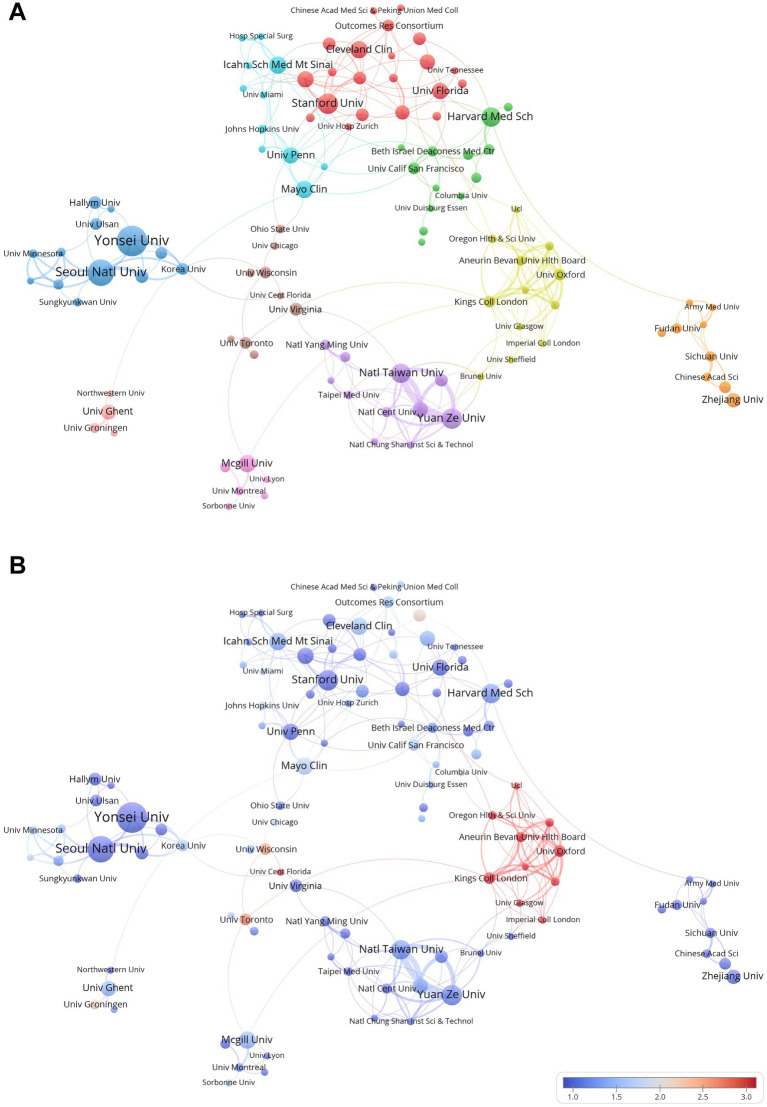
Institutional collaboration network in AI research in anesthesiology. **(A)** Co-occurrence network of research institutions in AI research (node size represents collaboration frequency; links indicate co-occurrence relationships). **(B)** Heatmap of recent publication activity in institutional collaborations (based on **A**, different shades represent the frequency of recent publication volume).

Figure 5 shows a collaboration map of major institutions. The institutions are grouped into 10 clusters based on their cooperation, with geographical patterns observed. For example, the purple cluster includes institutions from Taiwan, the light blue cluster represents U.S. institutions, and the orange cluster consists mostly of Chinese institutions. Notably, there are transnational collaborations, such as the red cluster with U.S. and Chinese institutions, and the yellow cluster involving institutions from England and the U.S. The blue cluster, dominated by South Korean institutions, includes the University of Minnesota System from the U.S. Figure 5B shows the strength of cooperation between structures, and it can be seen that there is a strong cooperation relationship within the yellow cluster, which is mainly composed of British institutions in Figure 5A, possibly indicating that these institutions have resources to contribute or complementary research advantages.

### 3.5 Journal analysis

Table 4 presents the top 10 journals with the highest number of publications and citations. *Anesthesia and Analgesia* leads with the highest publication volume (57 papers) and ranks second in citations (1,381), reflecting its authority in the field. *Anesthesiology*, despite fewer publications (18), has the highest citation count (1,459), indicating its significant academic recognition. Other key journals include the *British Journal of Anaesthesia* (40 publications, 1,181 citations).

**Table 4:** Top 10 journals in AI research in anesthesiology.

Rank	Journal	Publications	IF (JCR2023)	JCR quartile	Co-cited-journal	Citations	IF (JCR2023)	JCR quartile
1	Anesthesia and Analgesia	57	4.6	Q1	Anesthesiology	1,459	9.1	Q1
2	British Journal of Anaesthesia	40	9.1	Q1	Anesthesia and Analgesia	1,381	4.6	Q1
3	Bmc Anesthesiology	39	2.3	Q2	British Journal of Anaesthesia	1,181	9.1	Q1
4	Journal of Clinical Monitoring and Computing	33	2.0	Q2	Anaesthesia	456	7.5	Q1
5	Journal of Clinical Anesthesia	29	5.0	Q1	Acta Anaesthesiologica Scandinavica	272	1.9	Q2
6	Journal of Cardiothoracic and Vascular Anesthesia	24	2.3	Q2	Journal of Cardiothoracic and Vascular Anesthesia	243	2.3	Q2
7	Anesthesiology	18	9.1	Q1	Journal of Clinical Anesthesia	230	5.0	Q1
8	Journal of Anesthesia	15	2.8	Q2	Regional Anesthesia and Pain Medicine	224	5.1	Q1
9	European Journal of Anaesthesiology	12	4.2	Q1	Journal of Clinical Monitoring and Computing	197	2.0	Q2
10	Regional Anesthesia and Pain Medicine	12	5.1	Q1	New England Journal of Medicine	186	96.2	Q1

**Figure 6:**
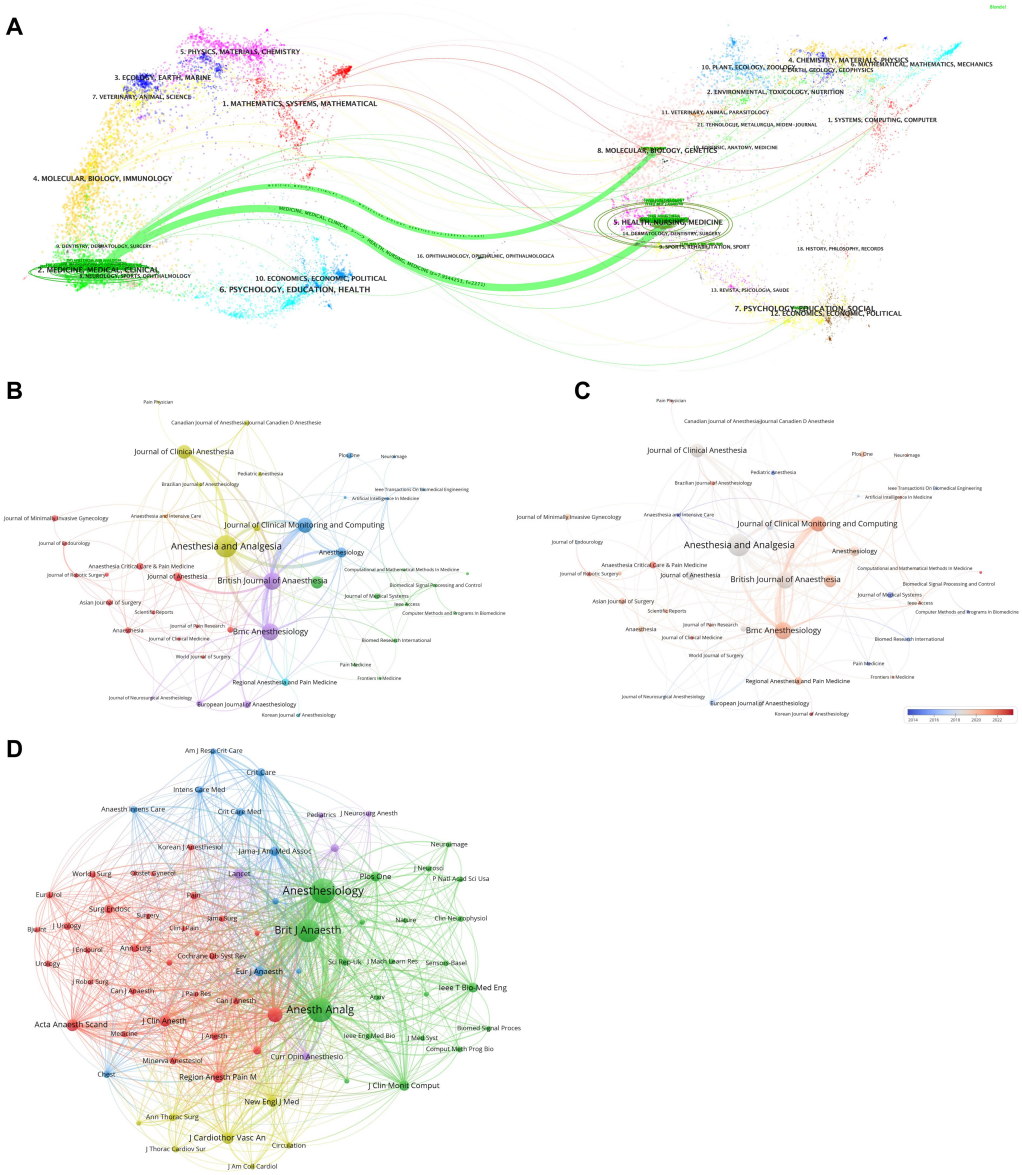
Network visualization of journal publications, collaboration, and citations in AI research in anesthesiology. **(A)** Dual-map overlay of citing and cited journals in AI research (citing journals on the left, cited journals on the right; colored trajectories indicate citation relationships). **(B)** Collaboration network of journals in AI research (different clusters distinguished by color, node size represents publication frequency). **(C)** Temporal influence analysis of journals in AI research (based on **B**; red indicates increasing influence, blue indicates declining activity). **(D)** Co-citation network of journals in AI research (node size represents citation frequency; links indicate co-citation relationships, highlighting journal influence).

Figure 6A displays a dual-mapping diagram, showing that clinical journals in AI in anesthesiology increasingly cite journals in the fields of Health, Nursing, and Medicine, as well as Molecular Biology and Genetics. This trend highlights the growing interdisciplinary nature of the research. It is evident that the focus of research is shifting from basic to clinical applications, which aligns with the ultimate goal of AI in anesthesiology. Co-occurrence and co-citation maps (Figures 6B–D) reveal strong collaborations, especially among journals like *Anesthesia and Analgesia*, *Journal of Clinical Monitoring and Computing*, *British Journal of Anaesthesia* and *Bmc Anesthesiology*.

To further elucidate the thematic structure of interdisciplinary research in AI and anesthesiology, we conducted a keyword cluster analysis based on journal classification. Specifically, we selected journals with ≥3 publications and categorized them into two major groups: anesthesiology/pain medicine journals and engineering/AI journals. Among the 658 articles, 452 (68.7%) were published in anesthesiology/pain-related journals, 82 (12.5%) in engineering/AI journals, and the remaining 124 (18.8%) across other specialties such as surgery, pediatrics, and orthopedics (Supplementary Figure 3A).

In anesthesiology/pain journals, six major thematic clusters were identified (Supplementary Figure 3B), including: robotic-assisted surgery, perioperative care, pain management, regional anesthesia, cardiothoracic anesthesia, and neuroanesthesia. These clusters reflect how clinical journal publications are closely aligned with practical anesthetic challenges, particularly emphasizing safety, effectiveness, and perioperative optimization.

In contrast, engineering/AI journals revealed four dominant clusters (Supplementary Figure 3C), including: perioperative hypotension prediction, EEG-based monitoring, airway and respiratory management, and pharmacologic modeling. These clusters highlight a methodological orientation, with a focus on data processing, signal analysis, and predictive modeling.

This comparative analysis underscores the dual progression of AI research in anesthesiology—from algorithmic development in engineering journals to translational clinical application in anesthesiology journals—revealing how foundational technological innovations increasingly support real-world anesthetic care.

### 3.6 Keywords analysis

**Figure 7:**
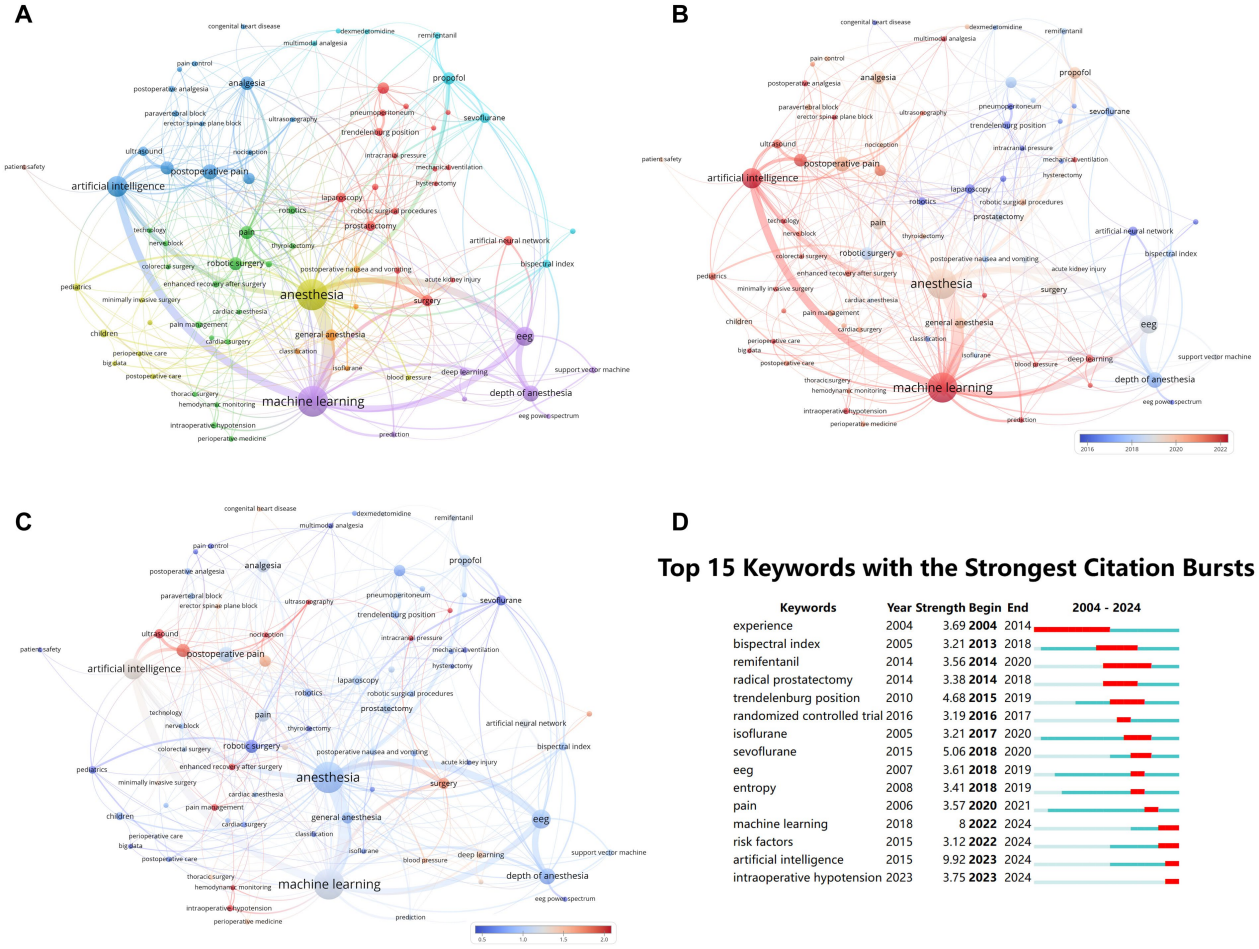
Keywords co-occurrence network in AI research in anesthesiology. **(A)** Co-occurrence network of keywords in AI research (nodes represent keyword clusters; size indicates co-occurrence frequency; links depict relationships among keywords). **(B)** Temporal trends in keyword influence (red indicates rising influence, blue indicates declining attention; color scale reflects recent keyword impact). **(C)** Heatmap of recent keyword attention (based on **B**; different shades indicate varying levels of recent research focus). **(D)** Keywords with citation bursts over time (top 15 keywords with sudden citation surges, marked by red spikes on the timeline).

Table 5 lists the 20 most frequent keywords in AI in anesthesiology. “anesthesia” (90 occurrences) and “machine learning” (87 occurrences) are the most prominent, with “artificial intelligence” (45 occurrences) trailing behind. These two keywords also show the highest link strength, reflecting their central role in current research. Co-occurrence and density visualizations (Figures 7A–C) reveal that these core terms are embedded within seven interconnected thematic clusters: Cluster 1 includes “artificial intelligence,” “postoperative pain,” and “ultrasound,” focusing on anesthesiology, pain management, imaging, and AI applications in healthcare. Cluster 2 includes “anesthesia,” “postoperative,” and “big data,” highlighting topics in anesthesiology, pediatrics, and postoperative care. Cluster 3 includes “EEG,” “machine learning,” and “support vector machine,” reflecting computational and data-driven approaches in medicine. Cluster 4 includes “pain,” “enhanced recovery after surgery,” and “nerve block,” associated with pain control, robotic surgery, and enhanced recovery protocols. Cluster 5 includes “trendelenburg position,” “artificial neural network,” and “prostatectomy,” focusing on surgical techniques and AI integration. Cluster 6 includes “propofol,” “sevoflurane,” and “multimodal analgesia,” centered on anesthetics and multimodal pain management. Cluster 7 includes “general anesthesia,” “postoperative nausea and vomiting,” and “isoflurane,” related to general anesthesia and common postoperative complications.

**Table 5:** Top 20 keywords in AI research in anesthesiology.

Rank	Keyword	Occurrences	Total link strength	Rank	Keyword	Occurrences	Total link strength
1	Anesthesia	90	144	11	Propofol	18	37
2	Machine learning	87	130	12	General anesthesia	16	22
3	Artificial intelligence	45	66	13	Opioid	16	25
4	EEG	39	67	14	Robot-assisted laparoscopic prostatectomy	16	22
5	Depth of anesthesia	29	44	15	Surgery	15	31
6	Postoperative pain	26	31	16	Prostatectomy	13	26
7	Analgesia	21	35	17	Sevoflurane	13	25
8	Pain	20	36	18	Laparoscopy	12	27
9	Regional anesthesia	20	35	19	Artificial neural network	11	12
10	Robotic surgery	20	23	20	Ultrasound	11	17

These clusters not only reflect research domains but also correspond to key perioperative stages—such as preoperative risk stratification, intraoperative guidance, and postoperative recovery—highlighting the practical anchoring of AI developments to real clinical workflows.

Figure 7D further supports these observations through burst detection analysis. Both “machine learning” and “artificial intelligence” show prolonged and intense burst periods through 2024, underscoring their sustained impact. Other keywords with burst durations that extend to 2024 include “intraoperative hypotension” and “risk factors,” suggesting that these keywords have received industry recognition and sustained attention.

### 3.7 Highly co-cited references analysis

**Figure 8:**
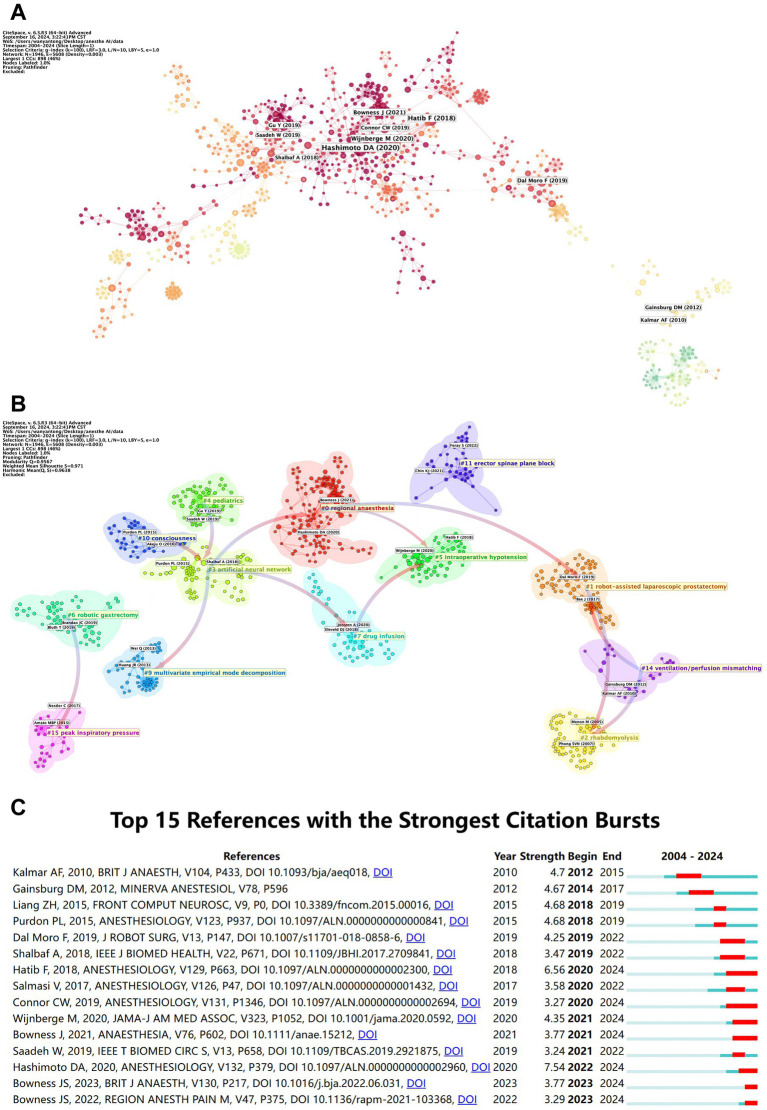
Co-citation network of highly cited references in AI research in anesthesiology. **(A)** Co-citation network of highly cited references (node colors represent publication years; node size indicates citation frequency). **(B)** Keyword heatmap and co-citation clusters (smaller numbers indicate larger clusters, with #0 as the largest; node size represents co-citation frequency, and links depict co-citation relationships). **(C)** References with citation bursts over time (top 15 references with sudden citation surges, marked by red spikes on the timeline).

Table 6 and Figure 8A shows that articles such as “Artificial Intelligence in Anesthesiology (9)” have garnered significant attention, indicating key research milestones in the field. Figure 8B groups these highly cited papers into 18 research clusters, covering topics like regional anesthesia, drug infusion, and robotic gastrectomy, reflecting the evolving focus of AI research in anesthesiology. Early research on topics like #10 consciousness and #4 pediatrics has influenced later advancements in #3 artificial neural networks and #9 multivariate empirical mode decomposition. Figure 8C shows citation bursts for the top articles. “Machine-learning Algorithm to Predict Hypotension (8)” and “Artificial Intelligence and Machine Learning in Anesthesiology (1)” have sustained attention, indicating their ongoing relevance in the field.

**Table 6:** Top 10 co-cited references in AI research in anesthesiology.

Rank	Article title	Journal	Co-citations	Centrality	Year
1	Artificial Intelligence in Anesthesiology: Current Techniques, Clinical Applications, and Limitations	Anesthesiology	24	0.07	2020
2	Machine-learning Algorithm to Predict Hypotension Based on High-fidelity Arterial Pressure Waveform Analysis	Anesthesiology	18	0.03	2018
3	Effect of a Machine Learning–Derived Early Warning System for Intraoperative Hypotension vs. Standard Care on Depth and Duration of Intraoperative Hypotension During Elective Noncardiac Surgery	Journal of the American Medical Association	13	0.02	2020
4	Artificial intelligence for image interpretation in ultrasound-guided regional anaesthesia	Anaesthesia	13	0.07	2021
5	Ultrasound-guided transversus abdominis plane block (US-TAPb) for robot-assisted radical prostatectomy: a novel “4-point” technique-results of a prospective, randomized study	Journal of Robotic Surgery	10	0.01	2019
6	Use of Multiple EEG Features and Artificial Neural Network to Monitor the Depth of Anesthesia	Sensors (Basel)	9	0.01	2019
7	Anesthetic concerns for robotic-assisted laparoscopic radical prostatectomy	Minerva Anestesiologica	9	0.12	2012
8	Monitoring the Depth of Anesthesia Using a New Adaptive Neurofuzzy System	IEEE Journal of Biomedical and Health Informatics	9	0.11	2018
9	Artificial Intelligence and Machine Learning in Anesthesiology	Anesthesiology	9	0.05	2019
10	Relationship between Intraoperative Hypotension, Defined by Either Reduction from Baseline or Absolute Thresholds, and Acute Kidney and Myocardial Injury after Noncardiac Surgery: A Retrospective Cohort Analysis	Anesthesiology	8	0.09	2017

## 4 Discussion

### 4.1 Publication and citation

Since the first publication in 1986 (18), research on AI in anesthesiology has grown slowly from 2004 to 2024. A significant turning point occurred between 2019 and 2020, nearly 8 years after the rapid surge in interest in AI within general medicine that began in 2012 (17). The synergy between AI advancements, particularly in machine learning and deep learning, and the expanding availability of large-scale clinical datasets has undeniably driven this growth. As the potential of AI in anesthesiology continues to be explored, publication output is expected to rise accordingly. The convergence of AI with real-time clinical data and predictive models represents a growing frontier that will likely see new and transformative applications in the near future.

### 4.2 Countries/regions, author and institution analysis

Since John McCarthy and other American scientists introduced the concept of using computers to simulate human intelligence, the United States has remained a global leader in this field. Other countries like South Korea and China have made significant contributions but tend to have more localized collaborations, primarily with the United States. The close collaborations between the United States and other countries such as South Korea are likely driven by shared research interests and geopolitical ties, which help foster deeper engagement in AI-related research. High-income countries have been the driving force behind AI research related to anesthesiology. The top 10 most productive countries are also ranked among the top 25 globally in terms of gross domestic product, suggesting that a nation’s economic capacity correlates with research productivity. This finding aligns with the results of bibliometric studies in various other medical fields (6, 19, 20).

The author analysis underscores the significant role of prolific authors such as Shieh, Jiann-Shing, Fan, Shou-Zen, and Abbod, Maysam F. in advancing AI research in anesthesiology. The strong co-citation frequency of authors like Myles and Hemmerling indicates their high academic influence and the relevance of their work in shaping the field. The co-citation relationship map reveals that many authors are involved in interdisciplinary research, particularly in the application of engineering and computer science to anesthesiology. The analysis of author cooperation and co-citations provides valuable insights into the academic dynamics of AI in anesthesiology and suggests that collaborative efforts, particularly those that bridge disciplines like neurology, engineering, and medicine, will likely drive future advancements in the field. Continuing to monitor the development of recently collaborating clusters, such as the Turan and Sessler group, will be essential for understanding emerging research trends.

The institution analysis highlights the leading role of South Korea in AI research in anesthesiology, with Yonsei University and Seoul National University ranking high both in publications and citations. U.S. institutions, such as Stanford University and Mayo Clinic, also contribute substantially to both publication volume and citation frequency, further emphasizing the dominance of U.S.-based research in this field. The collaboration map in Figure 5 reveals a strong regional clustering with notable cross-border collaborations, particularly between U.S., South Korean, and Chinese institutions. Among the top 10 institutions by publication volume, six are ranked in the top 100 of the QS World University Rankings, indicating that AI in anesthesiology has attracted the attention of leading global universities. Adequate research funding, diverse research partners, and a high proportion of foreign graduate students or visiting scholars are all key factors that promote international collaboration. Additionally, the sharing of biomedical data facilitates the development of AI in healthcare and promotes international collaboration (21). The development of AI is beneficial for research on healthcare interventions in low-and middle-income countries (22). However, British institutions demonstrate a higher level of institutional synergy, which may be attributed to structured research funding policies and well-established academic networks, enabling more effective collaborations.

### 4.3 Journal, keywords and highly co-cited references analysis

Among the top 10 high-output journals, 6 (60%) are classified as Q1 in the JCR, highlighting that AI research in anesthesiology is favored by leading journals in both anesthesiology and medical informatics. Examples include *Anesthesia and Analgesia*, *British Journal of Anaesthesia*, *Journal of Clinical Anesthesia*, and *Anesthesiology*. Furthermore, the top four co-cited journals are all high-impact Q1 publications, such as *Anesthesiology*, underscoring their significant role in advancing research in this field. Beyond citation metrics, our journal-based clustering analysis reveals a dual trajectory of AI research in anesthesiology—evolving from algorithmic development to clinical application. While anesthesiology journals primarily reflect urgent clinical needs and real-world adoption contexts, engineering and AI journals contribute foundational innovations in algorithms and modeling. Thematically, this clustering further validates the three key domains highlighted earlier—perioperative risk prediction, ultrasound-assisted anesthesia, and intelligent monitoring—reinforcing their centrality across both technological and clinical dimensions. These findings emphasize the translational significance and interdisciplinary integration of AI applications in anesthetic practice.

Intraoperative hypotension (IOH) is one of the most common complications during surgery and a known risk factor for increased postoperative myocardial injury, acute kidney injury, and mortality (23). The Hypotension Prediction Index (HPI), one of the first publicly available and clinically validated AI applications, is a machine learning algorithm that predicts hypotensive events by analyzing pulse wave contours with high fidelity. It has demonstrated good performance in predicting hypotension and reducing its duration (8, 24). However, the HPI may not be able to identify certain specific triggers of hypotension (25), such as obstructive shock, massive pneumothorax, pulmonary embolism, cardiac tamponade, or severe fluctuations due to surgical manipulation, rapid administration of anesthetics, and other factors. Additionally, other AI models, particularly those based on deep learning methods, have shown strong potential in predicting IOH. However, their ability to reduce IOH-related metrics, such as duration, remains unclear (24). In addition to predicting hypotension, AI models have been developed to predict a range of other complications, including acute kidney injury, delirium, myocardial injury, transfusion needs, hypoxemia, and mortality (26).

AI-driven ultrasound tools, such as the Kosmos system, ScanNav Anatomy Peripheral Nerve Block, and Nerveblox, are revolutionizing regional anesthesia procedures by enhancing the accuracy and efficiency of identifying anatomy and needle guidance (27). The use of AI for ultrasound-guided procedures is still in its early stages, but with the ongoing advancements in technology, these tools have shown promising results. For instance, ScanNav has demonstrated up to 95–100% accuracy in identifying anatomical structures during regional anesthesia procedures, suggesting its potential for widespread clinical adoption (28). Accurate needle placement is crucial for reducing complications and improving anesthetic efficacy, making AI-assisted ultrasound a promising innovation.

However, challenges remain in applying AI to deeper anatomical structures, such as the subgluteal sciatic nerve, or in situations where large insertion angles are required. While neural networks and machine learning have been employed to identify paravertebral and epidural structures (29, 30), further research is necessary to improve the accuracy and usability of these technologies for deep nerve blocks. Enhancing AI algorithms to handle complex cases, such as patients with significant anatomical variations or challenging procedural conditions, will be a crucial area of focus in future studies (31).

Despite these advances, several key research dimensions remain underrepresented in the current bibliometric landscape. Notably, concerns such as model interpretability, patient-centered outcomes, and multimodal data fusion—though increasingly discussed in general medical AI research—have not emerged as frequent keywords in anesthesiology-specific literature. Their absence underscores ongoing translational gaps that merit prioritization.

Although this study is bibliometric in nature, its findings reflect meaningful convergence between algorithmic innovation and clinical implementation. The identification of robust translational trends in perioperative risk prediction, AI-guided ultrasound, and anesthesia monitoring may help guide future research priorities toward more clinically integrated and patient-focused AI solutions.

## 5 Limitations

This study offers a comprehensive overview of research trends and hotspots in AI-related anesthesiology and highlights high-output countries/regions and academic institutions, facilitating potential collaborations and strategic research planning. However, there are several limitations to this research.

First, our study only included literature published in English, which means that important studies published in other languages may have been excluded from the analysis. Second, we restricted our analysis to the Web of Science database, which, while is a high-quality and rigorously indexed source, may not fully capture the breadth of research in the field.

Although the study provides valuable insights into publication trends and citation patterns, it does not assess the quality or clinical relevance of the individual studies. Citation counts, while an indicator of academic influence, do not necessarily reflect the true clinical impact of a given study. Additionally, the Web of Science database provides broad interdisciplinary coverage, encompassing fields such as medicine, life sciences, natural sciences, social sciences, and the humanities, making it ideal for cross-disciplinary research. The inclusion of other databases, such as Scopus, PubMed, and Google Scholar, could enhance coverage and provide a more comprehensive perspective on the topic. Lastly, we excluded papers published in formats such as book chapters, conference proceedings, papers, letters, news items, and corrections. As a result, relevant studies in these forms may have been overlooked.

Despite these limitations, our study provides a robust foundation for understanding the development and future directions of AI research in anesthesiology. Future research should aim to expand the scope of database selection, incorporate multilingual studies, and integrate more qualitative assessments to enhance the comprehensiveness of bibliometric analyses in this field.

## 6 Conclusion

Our study provides a comprehensive overview of AI research trends in anesthesiology, based on an analysis of 658 publications from the Web of Science database. Over the past 5 years, the number of AI-related publications in anesthesiology has grown rapidly, a trend that is projected to continue. This surge highlights the increasing significance of AI-driven innovations in anesthesiology, indicating that the field is gaining strong research momentum and is poised for further advancements.

We examined the countries and academic institutions with the highest research output, particularly focusing on those with strong international collaborations, such as the United States, China, and South Korea. The interdisciplinary nature of AI research in anesthesiology is clearly evident, as shown by the robust co-citation networks and the integration of engineering, computer science, and medical knowledge. As these research clusters continue to evolve, tracking emerging collaborations will be crucial for understanding the future trajectory of the field. Leading journals such as *Anesthesia* and *Analgesia and Anesthesiology* play a central role in disseminating pivotal AI research, further solidifying their influence in advancing AI applications within anesthesiology.

Keyword analyses reveal that “anesthesia” and “machine learning” dominate the literature, while emerging topics like “intraoperative hypotension” and “ultrasound” signal shifts toward clinically relevant AI applications. With ongoing advances in deep learning and real-time analytics, the development of AI systems capable of supporting intraoperative decision-making will be pivotal. Interdisciplinary collaboration will be essential to ensure these systems are not only technologically robust but also clinically safe and effective.

In conclusion, AI in anesthesiology is a rapidly evolving, interdisciplinary domain with strong translational potential. Continued research, clinical validation, and cross-disciplinary cooperation will be crucial in unlocking the full potential of AI to transform anesthetic care into a more precise, data-driven, and patient-centered practice.

## Data Availability

The original contributions presented in the study are included in the article/Supplementary material, further inquiries can be directed to the corresponding author.
