# Supplementary material for: Exploring the growth and impact of artificial intelligence in anesthesiology: a bibliometric study from 2004 to 2024
Source: Front Med (Lausanne). 2025 Jun 2;12:1595060. doi: 10.3389/fmed.2025.1595060 (PMC12171226; doi:10.3389/fmed.2025.1595060)
Supplement: Supplementary file 1 [file Data_Sheet_1.pdf]

Supplementary Figure 1. Trends in publications and citations of AI in anesthesiology (2004–2024). (A) Annual publication and citation trends in the field of AI in anesthesiology from 2004 to 2024. The number of publications (bars, left axis) and total citations (line, right axis) show a significant rise over the past five years, with a sharp increase in 2024. (B) Polynomial regression modeling based on annual publication data from 2004 to 2023 predicts a sustained growth trajectory. The exponential model fitted to the complete 2024 dataset (orange dashed line) further supports this trend, showing a steeper curve consistent with accelerating research momentum in the field.

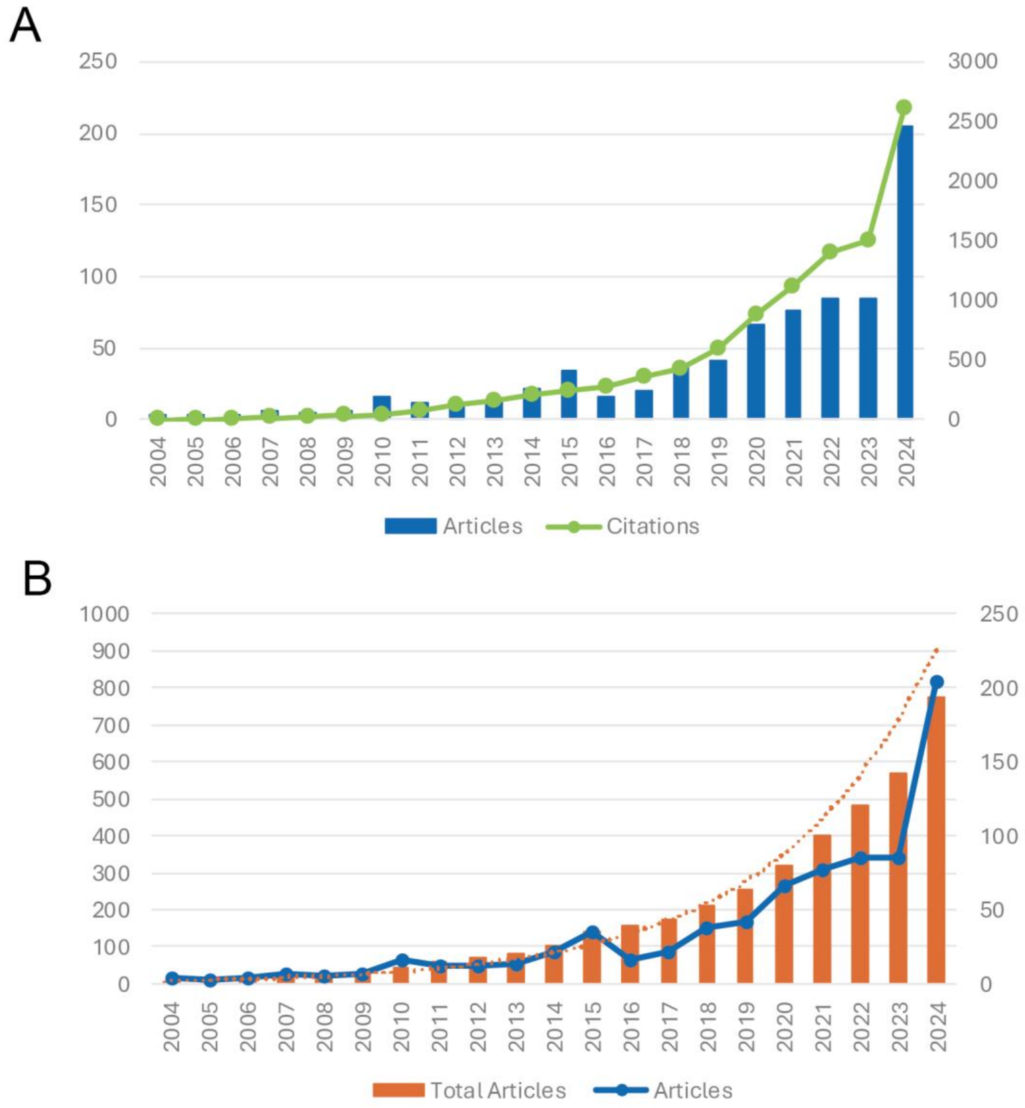

Supplementary Figure 2. Annual publication trends (2004–2024) in the field of AI in anesthesiology by country. The United States shows a consistently dominant growth trajectory, followed by China and South Korea. Other countries, including England, Germany, Japan, and Canada, display moderate but steadily increasing publication activity over the past decade.

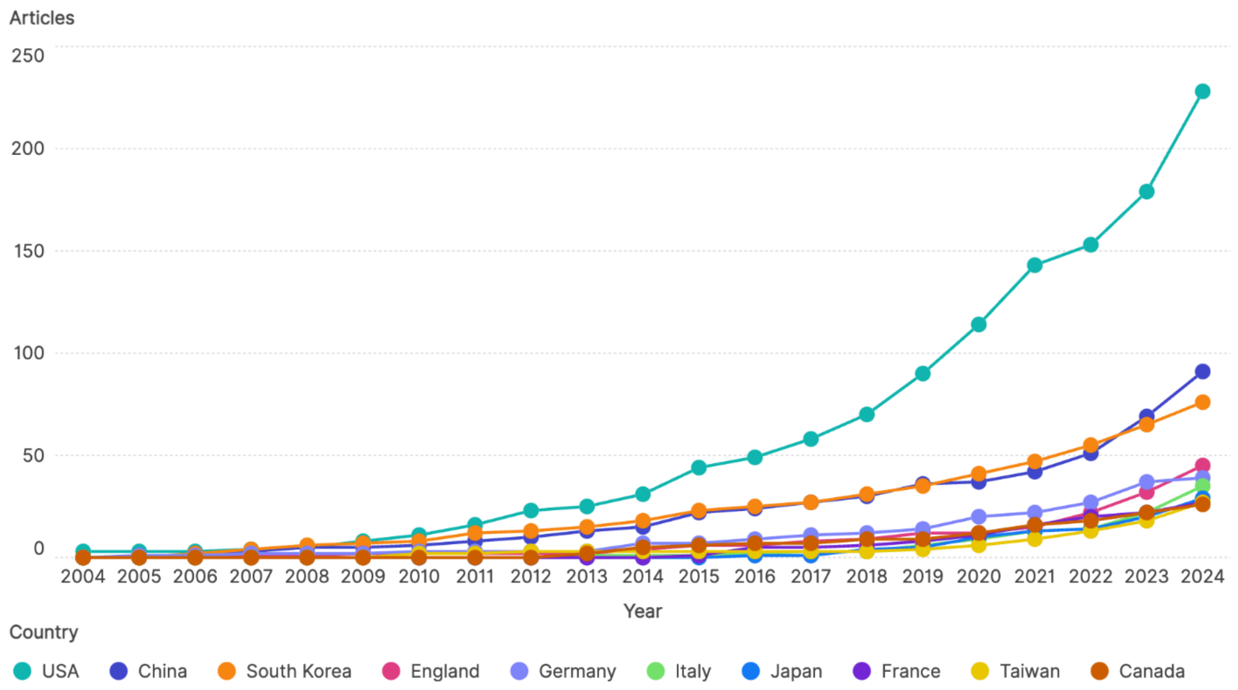

Supplementary Figure 3. Journal-based keyword clustering analysis of AI research in anesthesiology.

(A) Distribution of 658 articles by journal domain: 452 (68.7%) were published in anesthesiology/pain medicine journals, 82 (12.5%) in engineering/AI journals, and 124 (18.8%) in other specialties such as surgery and pediatrics.

(B) Keyword clustering from anesthesiology/pain journals revealed six major themes: robotic-assisted surgery (dark blue), perioperative management (light blue), regional anesthesia (red), pain management (green), cardiothoracic anesthesia (brown), and neuroanesthesia (mint green). These clusters reflect alignment with real-world clinical challenges in anesthesia.

(C) Keyword clustering from engineering/AI journals highlighted four dominant themes: perioperative hypotension (light blue), AI-driven electroencephalogram (EEG) analysis (green), perioperative airway management (red), and pharmacological management (dark blue), emphasizing methodological innovation and predictive modeling in anesthetic care.

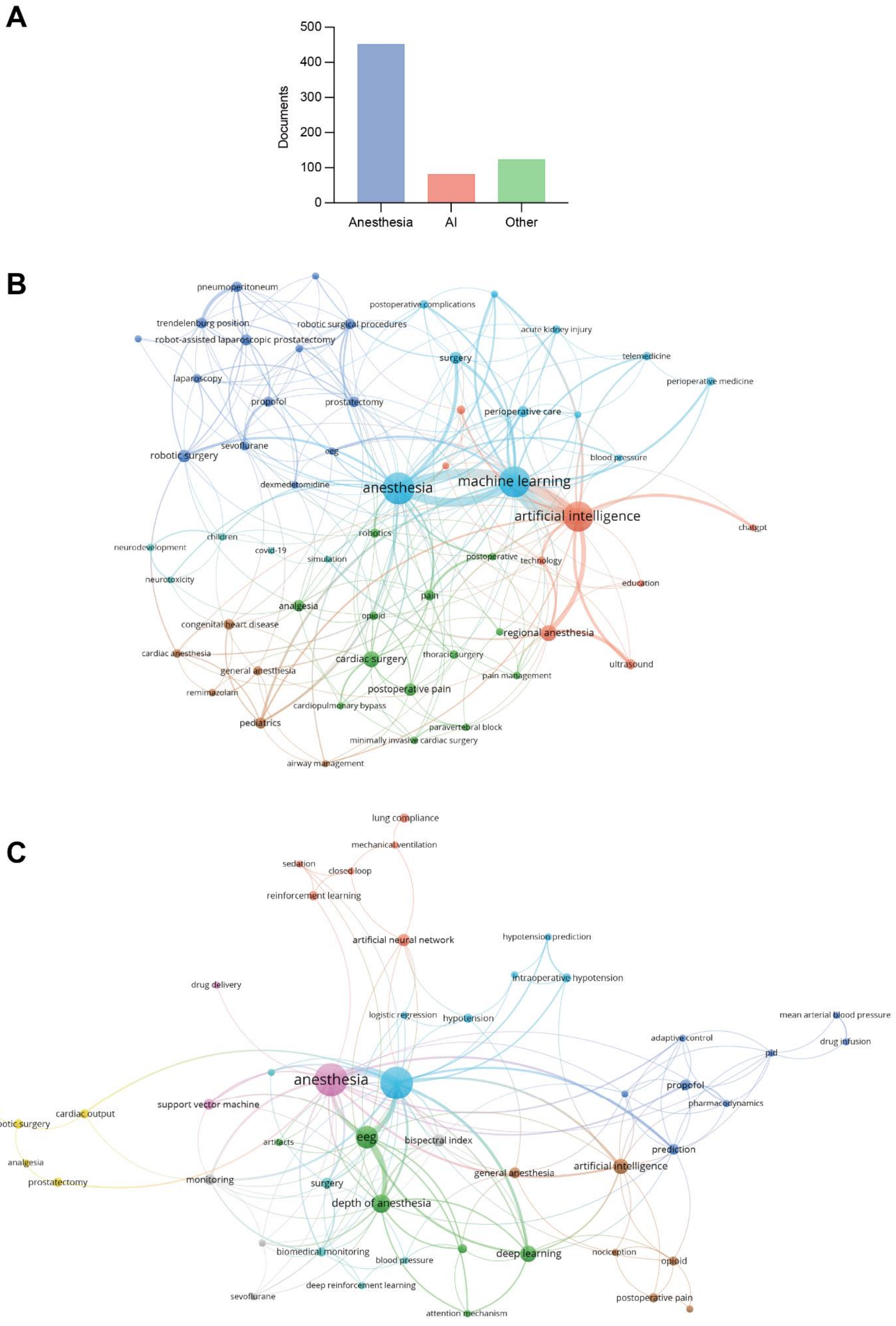

A
500
400
300
200
100
0
Documents
Anesthesia
AI
Other
B
pneumoperitoneum
trendelenburg position
robotic surgical procedures
robot-assisted laparoscopic prostatectomy
postoperative complications
acute kidney injury
telemedicine
surgery
perioperative medicine
laparoscopy
propofol
prostatectomy
perioperative care
sevoflurane
robotic surgery
eeg
blood pressure
dexmedetomidine
anesthesia
machine learning
artificial intelligence
chatgpt
robotics
children
neurodevelopment
covid-19
simulation
postoperative
technology
neurotoxicity
education
analgesia
pain
opioid
congenital heart disease
regional anesthesia
cardiac anesthesia
cardiac surgery
thoracic surgery
ultrasound
general anesthesia
pain management
remimazolam
postoperative pain
cardiopulmonary bypass
pediatrics
paravertebral block
minimally invasive cardiac surgery
airway management
C
lung compliance
mechanical ventilation
sedation
closed loop
reinforcement learning
artificial neural network
hypotension prediction
drug delivery
intraoperative hypotension
logistic regression
hypotension
mean arterial blood pressure
adaptive control
drug infusion
pid
anesthesia
propofol
robotic surgery
cardiac output
support vector machine
pharmacodynamics
artifacts
eeg
bispectral index
prediction
analgesia
prostatectomy
monitoring
surgery
general anesthesia
artificial intelligence
depth of anesthesia
biomedical monitoring
blood pressure
deep learning
nociception
opioid
deep reinforcement learning
sevoflurane
postoperative pain
attention mechanism
